# Nutrient-Induced Remodeling of the Adipose-Cardiac Axis: Metabolic Flexibility, Adipokine Signaling, and Therapeutic Implications for Cardiometabolic Disease

**DOI:** 10.3390/nu17243945

**Published:** 2025-12-17

**Authors:** Nikola Pavlović, Petar Todorović, Mirko Maglica, Marko Kumrić, Joško Božić

**Affiliations:** 1 Department of Pathophysiology, University of Split School of Medicine, 21000 Split, Croatia; 2 Laboratory for Cardiometabolic Research, University of Split School of Medicine, 21000 Split, Croatia; 3 Department of Anatomy, Histology and Embryology, University of Split School of Medicine, 21000 Split, Croatia; 4 Department of Anatomy, School of Medicine, University of Mostar, 88000 Mostar, Bosnia and Herzegovina; 5 Department of Cardiovascular Diseases, University Hospital of Split, 21000 Split, Croatia

**Keywords:** metabolic syndrome, adipose tissue phenotypes, nutrient-sensing pathways, cardiometabolic dysfunction, precision nutrition

## Abstract

Insulin resistance, dyslipidemia, hypertension, and visceral adiposity are the leading causes of the growing worldwide health burden associated with metabolic syndrome, obesity, and cardiovascular diseases (CVDs). Despite the “obesity paradox,” which emphasizes the varied cardiovascular outcomes among obese people, obesity is now acknowledged as an active contributor to cardiometabolic dysfunction through endocrine, inflammatory, and metabolic pathways. Growing evidence indicates that nutrition is a key determinant of cardiometabolic risk, highlighting the need to understand diet-mediated mechanisms linking adipose tissue to cardiac function. Adipokines, including adiponectin, leptin, TNF-α, and resistin, which regulate systemic inflammation, metabolic homeostasis, and myocardial physiology, are secreted by adipose tissue, which is no longer thought of as passive energy storage. Its heterogeneous phenotypes, white, brown, and beige adipose tissue, exhibit distinct metabolic profiles that influence cardiac energetics and inflammatory status. Nutrient-driven transitions between these phenotypes further underscore the intricate interplay between diet, adipose biology, and cardiac metabolism. Central nutrient-sensing pathways, including mTOR, AMPK, SIRT1, PPAR-γ, and LKB1, integrate macronutrient and micronutrient signals to regulate adipose tissue remodeling and systemic metabolic flexibility. These pathways interact with hormonal mediators such as insulin, leptin, and adiponectin, forming a complex regulatory network that shapes the adipose-cardiac axis. This review synthesises current knowledge on how nutrient inputs modulate adipose tissue phenotypes and signaling pathways to influence cardiac function. By elucidating these mechanisms, we highlight emerging opportunities for precision nutrition and targeted therapeutics to restore metabolic balance, strengthen cardiac resilience, and reduce the burden of cardiometabolic disease.

## 1. Introduction

Cardiovascular diseases (CVDs), obesity, and metabolic syndrome together constitute a major worldwide health concern that dramatically raises rates of morbidity and mortality [1]. Up to 65% of patients presenting with acute coronary syndrome in some populations have metabolic syndrome, according to recent epidemiological investigations that indicate concerning prevalence rates globally [2,3]. Insulin resistance, hypertension, increased blood lipids (dyslipidemia), and abdominal obesity are the main causes of this epidemic. With distinct processes connecting signals from adipose tissue to heart dysfunction, obesity has been found to be an independent and controllable risk factor for CVD [4]. Nevertheless, the scientific community also acknowledges the so-called “obesity paradox”, unexpected findings that suggest some obese individuals maintain relatively preserved heart function and a lower mortality risk [5,6]. Despite these exceptions, dietary interventions remain pivotal in modifying cardiovascular risk, thereby underscoring the fundamental role of nutrition in both disease prevention and treatment [7,8].

The understanding of adipose-cardiac interactions has shifted from viewing adipose tissue merely as a passive energy store to recognising its active endocrine function. The discovery of adipokines such as adiponectin, tumour necrosis factor-alpha (TNF-α), leptin, and resistin marked a paradigm shift, revealing how adipose tissue dysfunction contributes to systemic inflammation and cardiac pathology [9,10]. This interdisciplinary insight transformed perspectives from organ-centric pathophysiology to integrative, system-level frameworks encompassing metabolic, inflammatory, and neurohumoral pathways that interlink adipose tissue and cardiac function [11].

Adipose tissue exhibits heterogeneous phenotypes with distinct metabolic roles [12]. White adipose tissue (WAT) predominantly displays a metabolically inflexible and pro-inflammatory profile associated with lipid storage and insulin resistance. Brown adipose tissue (BAT), conversely, is thermogenic, metabolically flexible, and exerts anti-inflammatory effects through oxidative metabolism and non-shivering thermogenesis. Beige adipose tissue exhibits an intermediate phenotype, capable of WAT-to-BAT-like transitions in response to nutrient and hormonal stimuli [13,14]. These nutrient-mediated phenotypic shifts have crucial implications for cardiac metabolism, influencing substrate availability and inflammatory milieu in the adipose-cardiac axis [15].

Adipose tissue integrates nutrient signals through master regulators such as mechanistic target of rapamycin (mTOR), AMP-activated protein kinase (AMPK), sirtuin-1 (SIRT1), peroxisome proliferator-activated receptor gamma (PPAR-γ), and liver kinase B1 (LKB1) [12,16]. Macronutrients differentially modulate these pathways—glucose primarily activates mTOR, amino acids engage mTOR and related sensors, and lipids influence PPAR-γ activity [17]. Micronutrients serve as essential cofactors for energy-metabolism enzymes, further refining the cellular response to nutrient availability. This signaling network interfaces with endocrine hormones, including insulin, leptin, and adiponectin, orchestrating metabolic flexibility and systemic energy balance [18,19].

While recent reviews have examined individual aspects of adipose-cardiac communication, metabolic flexibility, or dietary effects on cardiovascular health, an integrative framework connecting these elements remains lacking. Current literature often addresses macronutrient and micronutrient effects in isolation, without linking dietary inputs to the full spectrum of adipose phenotypes and their cardiac consequences. Furthermore, emerging mediators such as adipose-derived extracellular vesicles and gut microbiota metabolites are rarely discussed alongside classical adipokine pathways. This review addresses these gaps by synthesizing nutrient-sensing mechanisms, adipose tissue remodeling, and inter-organ communication within a unified framework that bridges molecular insights with clinical biomarkers and therapeutic targets.

Understanding the adipose-cardiac axis is vital for precision medicine approaches targeting cardiometabolic diseases. Current knowledge gaps persist regarding how different nutrients and dietary patterns remodel this axis at the molecular and systemic levels. This review aims to elucidate these processes and their implications for developing targeted nutritional and pharmacological interventions to restore metabolic balance, enhance cardiac resilience, and reduce disease burden.

## 2. Molecular Mechanisms of Nutrient Sensing and Signaling in Adipose Tissue and Heart

The molecular mechanisms that govern nutrient sensing and signaling across adipose tissue and the heart form an integrated regulatory network essential for maintaining metabolic homeostasis [20]. Adipose tissue acts as both a nutrient reservoir and an endocrine organ, translating fluctuations in energy availability into cellular and systemic responses. These processes are shaped by the anatomical distribution of adipose depots, subcutaneous, visceral, perivascular, and epicardial, which differ in metabolic activity, vascularization, innervation, and cellular composition [21]. Within each depot, adipocytes interact with stromal vascular cells and immune populations to coordinate nutrient uptake, lipid storage, and endocrine output. Through the synthesis and secretion of adipokines, adipose tissue communicates nutrient status to peripheral organs, including the heart, thereby influencing cardiac substrate selection, oxidative capacity, and inflammatory tone [22,23].

Among the adipokines involved in nutrient-dependent communication, adiponectin represents a key metabolic checkpoint [24]. Its circulating levels are determined by the molecular structure encoded by the ADIPOQ gene and by nutritional conditions that modulate its transcription and multimerization [25]. Adiponectin signals primarily through AdipoR1, AdipoR2, and T-cadherin, activating downstream pathways that enhance cellular energy efficiency. Central to its action is robust AMPK stimulation, which, in turn, activates PGC-1α, promoting mitochondrial biogenesis and improved oxidative metabolism. In cardiomyocytes, adiponectin facilitates glucose handling by enhancing GLUT4 translocation to the cell surface, while simultaneously increasing fatty acid oxidation capacity [26,27]. Beyond metabolic effects, adiponectin generates strong anti-inflammatory and antioxidative responses by suppressing NF-κB signaling and enhancing mitochondrial reactive oxygen species scavenging. Dietary composition directly influences adiponectin secretion, with unsaturated fats, fiber-rich diets, and caloric moderation promoting higher adiponectin levels and greater receptor responsiveness [28,29].

In contrast, nutrient excess and adipose tissue dysfunction lead to increased production of pro-inflammatory adipokines such as resistin, TNF-α, and IL-6 [30,31]. These cytokines exert both paracrine effects within adipose depots and systemic actions that reach the heart. By activating TLR4 and the NF-κB pathway in cardiac tissue, they promote oxidative stress, impair myocardial insulin signaling, and drive pathological remodeling, including hypertrophy and fibrosis [32,33]. In parallel, endothelial dysfunction arises from inflammatory signaling and elevated circulating lipids. High glucose load, saturated fatty acids, and nutrient oversupply are potent drivers of these pro-inflammatory secretory programs, linking dietary patterns to cardiometabolic dysfunction [34,35].

Leptin also plays a critical role in nutrient-derived signaling between adipose tissue and the heart [36,37]. Cardiomyocytes express functional leptin receptors through which leptin modulates substrate metabolism, fatty acid utilization, and myocardial contractility [38]. Under physiological conditions, leptin helps maintain metabolic balance; however, chronic energy surplus leads to leptin resistance, blunting its regulatory effects. As a result, cardiac tissue becomes exposed to persistent nutrient overload and inadequate metabolic adaptation, which contributes to lipotoxicity, impaired contractile function, and progression toward cardiometabolic disease [38,39,40].

Metabolic byproducts originating from adipose tissue provide an additional layer of nutrient-dependent communication [41,42]. Free fatty acids (FFAs) function both as essential nutrient signals and as lipotoxins when present in excess, influencing cardiac energy metabolism and mitochondrial integrity [43]. Glycerol, released during lipolysis, serves as a substrate for hepatic gluconeogenesis and indirectly affects cardiac substrate supply [44]. Ketone bodies, particularly β-hydroxybutyrate and acetoacetate, produced during enhanced lipolytic flux, are readily utilized by the heart as efficient oxidative fuels [45,46]. Moreover, adipose tissue releases extracellular vesicles enriched in microRNAs, such as miR-223 and miR-122, which modulate cardiac gene expression, metabolic pathways, and inflammatory signaling [47].

The heart integrates these diverse adipose-derived nutrient cues through a network of receptors and transcriptional regulators [16,41]. PPAR-α acts as the central transcriptional controller of fatty acid oxidation genes, adjusting myocardial substrate use in response to lipid availability [48]. PPAR-γ contributes to metabolic and anti-inflammatory regulation, whereas AMPK serves as the primary cellular energy sensor, responding to shifts in ATP demand and substrate supply. Sirtuins (SIRT1, SIRT3, SIRT7) link nutrient state to gene expression via NAD+-dependent deacetylation reactions, influencing mitochondrial function, redox status, and cardiac stress responses. AdipoR1 and AdipoR2, as key receptors for adiponectin, represent major integration points for nutrient-sensitive endocrine signaling, coordinating AMPK and PPAR pathways to optimize cardiac metabolic adaptation [49,50].

Collectively, these adipokine pathways do not operate in isolation but converge on shared intracellular nodes, particularly AMPK, mTOR, and NF-κB, where the balance between cardioprotective and cardiotoxic signals determines disease trajectory [51,52]. This mechanistic interdependence implies that targeting a single pathway may yield limited benefit if upstream adipose dysfunction persists. Multi-targeted strategies, including lifestyle modifications that simultaneously reduce pro-inflammatory adipokine secretion, enhance adiponectin output, and restore leptin sensitivity, are therefore more likely to achieve durable cardioprotection. These pathway interactions also inform biomarker development; composite panels reflecting both protective (adiponectin) and harmful (resistin, TNF-α, ceramides) signals may improve cardiovascular risk prediction beyond single markers [53].

Through these interconnected pathways, the heart “interprets” adipose-derived signals and adjusts its metabolic priorities. When nutrient sensing across tissues is balanced, this axis supports metabolic flexibility, efficient substrate use, and protection against oxidative and inflammatory stress. However, chronic nutrient excess, depot-specific adipose dysfunction, and endocrine imbalance disrupt this communication network, accelerating the transition from adaptive metabolic remodeling to cardiometabolic disease [12,51].

## 3. Dietary Patterns and Their Impact on Adipose Tissue and Cardiac Function

### 3.1. Mediterranean Diet (MedDiet)

The Mediterranean diet is characterized by a nutrient profile rich in monounsaturated fatty acids from olive oil, high-quality complex carbohydrates from whole grains, moderate protein intake with an emphasis on fish over red meat, abundant polyphenols from fruits and vegetables, and moderate wine consumption. These components synergistically modulate adipose tissue biology and cardiometabolic function [54,55,56].

Within adipose tissue, polyphenol-mediated PPAR-γ activation enhances insulin sensitivity and supports the maintenance of a metabolically healthy adipocyte phenotype. MedDiet adherence promotes the preservation and recruitment of brown adipose tissue (BAT) characteristics while reducing white adipose tissue (WAT) inflammation by inhibiting NF-κB [57,58]. A consistent finding across mechanistic and clinical studies is the upregulation of adiponectin, accompanied by the suppression of pro-inflammatory adipokines. These processes converge on increased mitochondrial biogenesis, primarily driven by PGC-1α activation, thereby improving oxidative capacity in adipocytes [59,60].

Cardiac tissue benefits from these systemic improvements through greater metabolic flexibility, enhanced endothelial function, and modulation of vascular tone. MedDiet interventions have been associated with reduced atrial fibrillation burden, improvements in diastolic function, particularly in HFpEF populations, and attenuation of prothrombotic pathways. The diet’s influence on cardiac autonomic regulation, reflected by increased parasympathetic and reduced sympathetic activity, further contributes to cardioprotection [61].

Polyphenols serve as central mediators of these effects. Resveratrol activates SIRT1, improving mitochondrial resilience to oxidative stress; catechins exhibit potent antioxidant and anti-inflammatory actions; and anthocyanins enhance endothelial nitric oxide production and vascular reactivity. These molecular actions correspond with observed reductions in LDL oxidation, improved SCORE2-based cardiovascular risk estimates, and enhanced endothelial-dependent vasodilation. Landmark trials such as PREDIMED and multiple meta-analyses consistently validate the MedDiet as one of the most effective dietary patterns for reducing cardiovascular morbidity and mortality [62,63,64,65].

### 3.2. Ketogenic Diet

The ketogenic diet imposes a profound metabolic shift characterized by carbohydrate restriction and reliance on ketone bodies derived from fatty acids. Increased adipose lipolysis produces elevated acetyl-CoA, which drives hepatic ketogenesis [66,67]. The heart efficiently oxidizes β-hydroxybutyrate, which can serve as a preferred oxidative substrate under ketogenic conditions. Alterations in the NADH/NAD+ ratio and increased reliance on fatty acid oxidation (FAO) constitute key metabolic adaptations [68,69].

In adipose tissue, ketogenic feeding enhances lipolytic capacity, stimulates BAT activation and beige adipocyte differentiation through β-3 adrenergic signaling, and reduces WAT mass alongside infiltration of pro-inflammatory immune cells. These changes translate into a more favorable adipokine profile, notably with increased adiponectin secretion [70,71]. Cardiac adaptations include enhanced mitochondrial efficiency, improved substrate switching, and reduced susceptibility to delayed afterdepolarizations (DADs) associated with calcium overload, thereby potentially lowering arrhythmogenic risk [72]. However, ketogenic diets may cause a rise in LDL-cholesterol, driven by increased lipoprotein turnover, reduced LDL receptor activity, and enhanced hepatic VLDL secretion. The clinical significance of this elevation varies between individuals and remains an area of ongoing investigation [73]. Despite short-term benefits, particularly weight loss and improved glycemic control, long-term adherence is challenging due to dietary monotony, nutrient deficiencies, increased risk of kidney stones, and socioeconomic constraints. These limitations underscore the importance of tailored patient selection and careful monitoring in clinical applications [74,75].

### 3.3. Intermittent Fasting (IF) and Time-Restricted Eating (TRE)

Intermittent fasting and time-restricted eating induce characteristic metabolic cascades centered on energy sensing. Fasting intervals activate AMPK and inhibit mTORC1, promoting autophagy, mitophagy, and enhanced lipid oxidation. These processes elevate circulating ketone bodies and improve mitochondrial quality across metabolic tissues [76,77]. In adipose tissue, IF fosters a shift toward a metabolically healthier phenotype, with increased BAT activation via sympathetic nervous system engagement and β-3 adrenergic signaling. Adipokine secretion profiles improve, and feeding-fasting cycles reinforce circadian rhythm alignment, which is essential for optimal adipose and systemic metabolic function [78,79]. Cardiac tissue similarly benefits from increased autophagy and improved mitochondrial turnover, along with reduced oxidative stress and systemic inflammation [80]. However, specific fasting protocols may predispose susceptible individuals to arrhythmias, potentially via autonomic shifts, electrolyte fluctuations, or ketone-mediated electrophysiological changes. Protocols such as 16:8, 5:2, and alternate-day fasting differ in metabolic impact and adherence profiles. Early time-restricted eating (TRE) protocols show superior effects on reducing visceral adipose tissue compared to late TRE, which is particularly important for cardiometabolic protection. However, the optimal fasting window varies by genotype and chronotype, highlighting the need for personalized fasting schedules [81,82]. Population-specific responses are increasingly recognized: women, older adults, and individuals with advanced metabolic impairment may require modified fasting windows to optimize safety and efficacy [83,84].

Chrononutrition, distinct from time-restricted eating, emphasizes the importance of synchronizing food intake with endogenous circadian rhythms, which are governed by peripheral clocks in adipose tissue, the heart, the liver, and other organs. Each individual has a unique optimal timing for food consumption aligned with these rhythms. Late meals, a common concern, can directly disrupt gene expression in adipose tissue and cardiac muscle, adversely affecting metabolic regulation and circadian alignment. Therefore, timing meals to align with one’s circadian biology is critical for maintaining metabolic homeostasis and preventing dysfunction.

### 3.4. Caloric Restriction (CR)

Caloric restriction (CR) induces a coordinated systemic metabolic response [85]. Reduced caloric intake lowers hepatic glucose production, enhances whole-body insulin sensitivity, and decreases circulating FFAs by modulating adipose lipolysis. In adipose tissue, CR leads to a reduction in WAT mass, improved insulin signaling, increased adiponectin levels, and decreased macrophage infiltration, all characteristics of a more insulin-sensitive and anti-inflammatory adipose phenotype [86,87,88]. Cardiac-specific benefits arise through activation of the SIRT1–PGC-1α axis, culminating in enhanced mitochondrial biogenesis and improved myocardial energetic capacity [89,90]. CR also augments endothelial function and nitric oxide availability, improves coronary flow reserve, and supports diastolic performance [91,92]. Redox homeostasis is a central feature of CR. Reduced caloric intake lowers electron flux through the mitochondrial electron transport chain, decreasing ROS generation. Concurrently, antioxidant enzyme expression increases, NAD+ availability rises, and SIRT1 activity is enhanced, jointly contributing to improved mitochondrial efficiency and stress resistance [93,94]. While CR reliably induces weight loss, its long-term feasibility is limited, and excessive restriction may jeopardize lean mass. Strategies incorporating resistance training, periodic refeeding, and protein adequacy can help preserve skeletal muscle while maintaining metabolic benefits [95].

### 3.5. DASH Diet (Dietary Approaches to Stop Hypertension)

The DASH diet emphasizes fruits, vegetables, whole grains, lean proteins, and low-fat dairy while limiting sodium, saturated fat, and added sugars. Originally developed for hypertension management, DASH has demonstrated broad cardiometabolic benefits extending to adipose tissue function and cardiac health [96,97]. Within adipose tissue, DASH reduces visceral adiposity and attenuates chronic low-grade inflammation by decreasing pro-inflammatory immune cell infiltration and suppressing NF-κB-mediated cytokine production. Clinical trials report significant reductions in circulating *C*-reactive protein, LDL-cholesterol, and markers of oxidative stress, accompanied by improvements in insulin sensitivity and adipokine profiles [98,99]. The diet’s high potassium, magnesium, and fiber content promotes vasodilation, enhances endothelial function, and favorably modulates the renin–angiotensin–aldosterone system. Cardiac benefits include cumulative reductions in subclinical myocardial injury (hs-cTnI) and systemic inflammation over sustained adherence periods, with meta-analyses demonstrating 6–11 mmHg reductions in systolic blood pressure independent of weight loss [100]. DASH shares anti-inflammatory characteristics with the Mediterranean diet but places greater emphasis on sodium restriction and dairy inclusion, making it particularly suitable for hypertensive populations at elevated cardiovascular risk.

### 3.6. Plant-Based Diets

Plant-based diets, encompassing vegetarian, vegan, and whole-food plant-based patterns, exert favorable effects on adipose tissue biology and cardiac function through multiple mechanisms [101,102]. High intake of dietary fiber, polyphenols, and antioxidants reduces oxidative stress and reactive oxygen species (ROS) generation in adipose tissue, thereby attenuating adipocyte hypertrophy and inflammatory adipokine secretion. Plant-based patterns consistently lower circulating leptin while increasing adiponectin, reflecting improved adipose tissue insulin sensitivity and reduced visceral fat accumulation [103]. The absence of dietary cholesterol and reduced saturated fat intake decrease hepatic VLDL secretion and improve lipid profiles, with vegan diets showing the most pronounced reductions in total cholesterol, LDL-cholesterol, and apolipoprotein B [104]. Cardiac benefits extend beyond risk factor modification: interventional studies in heart failure patients report improvements in ejection fraction, stroke volume, and cardiac remodeling following adoption of plant-based diets [105,106]. Mechanistically, reduced TMAO production (due to altered gut microbiota composition lacking TMA-producing bacteria), lower advanced glycation end-products (AGEs), and enhanced nitric oxide bioavailability contribute to improved endothelial function and reduced myocardial fibrosis. Plant-based diets also lower blood pressure through mechanisms similar to DASH, including increased potassium intake and favorable effects on vascular tone [107]. Potential limitations include the need for careful planning to ensure adequate intake of vitamin B12, iron, zinc, and omega-3 fatty acids, particularly in strictly vegan patterns.

### 3.7. Direct Inter-Diet Comparisons and Personalization Strategies

Direct comparisons of dietary patterns reveal distinct macronutrient compositions, caloric densities, adipose tissue effects, and cardiac outcomes. The MedDiet offers broad cardiometabolic benefits with high sustainability; ketogenic diets provide potent short-term metabolic switching at the cost of long-term adherence and variable lipid responses; IF/TRE protocols optimize circadian alignment and cellular repair, and CR delivers robust improvements in insulin sensitivity and mitochondrial function but is challenging to maintain chronically [76,85,108]. Personalization strategies should account for adipose phenotype (visceral vs. subcutaneous predominance, inflammatory state, BAT activity), metabolic status, comorbidities, and individual lifestyle factors. Nutrigenomics further refines these approaches by identifying genetic polymorphisms that modulate responses to dietary interventions like TRE or CR. For example, APOE variants influence lipid responses to Mediterranean diet patterns, with ε4 carriers showing greater cholesterol reductions from monounsaturated fat emphasis, while FTO rs9939609 polymorphism affects satiety signaling and body weight loss efficacy during intermittent fasting, particularly in obesity-prone individuals. MTHFR polymorphisms also impact metabolic responses to nutrient intake, underscoring genotype-specific diet optimization. Beyond genetic determinants, substantial inter-individual heterogeneity in dietary response arises from age, biological sex, baseline metabolic phenotype, gut microbiome composition, and physical activity level [109,110]. Older adults exhibit reduced metabolic flexibility and anabolic resistance that may blunt intervention efficacy; women and men differ in fat distribution and substrate oxidation, leading to sexually dimorphic responses; and individuals with greater baseline insulin resistance or adipose inflammation often show larger absolute improvements but may require more intensive protocols [111]. These multidimensional sources of variability underscore that optimal dietary prescription requires integration of genetic, phenotypic, demographic, and lifestyle factors toward truly personalized nutrition approaches. Synergistic approaches, such as combining MedDiet with TRE or cycling mild CR periods, may maximize metabolic flexibility and cardioprotection while maintaining adherence.

In summary, dietary patterns modulate the adipose-cardiac axis through distinct but overlapping mechanisms: the Mediterranean diet enhances adiponectin and PPAR-γ signaling; ketogenic diets shift fuel utilization toward ketone bodies and activate BAT; intermittent fasting promotes AMPK-driven autophagy and circadian alignment; and caloric restriction activates the SIRT1–PGC-1α axis. Selecting among these approaches should be guided by individual metabolic phenotype, comorbidities, and long-term adherence potential.

## 4. Role of Micronutrients in Adipose and Cardiac Metabolism

While the preceding sections have addressed macronutrients and dietary patterns, micronutrients serve as essential cofactors and regulators that intersect with the same molecular nodes—AMPK, SIRT1, NF-κB, and mitochondrial signaling pathways—that govern adipose-cardiac crosstalk [112,113]. Rather than functioning as isolated factors, micronutrients modulate the activity and expression of nutrient-sensing cascades discussed earlier, thereby amplifying or mitigating macronutrient-driven metabolic dysfunction. For example, vitamin D insufficiency compromises AMPK/SIRT1 activation in adipose tissue, whereas magnesium deficiency impairs mitochondrial ATP synthesis and promotes oxidative stress through shared pathways affected by caloric excess [114,115]. This mechanistic overlap underscores the importance of adequate micronutrient status in preserving the metabolic flexibility and anti-inflammatory balance that protect cardiac function.

Micronutrient status profoundly influences metabolic and inflammatory pathways in both adipose tissue and the heart. Deficiencies or excesses of certain vitamins and minerals can perturb adipokine secretion, insulin sensitivity, and mitochondrial function, thereby contributing to cardiometabolic diseases such as obesity, type 2 diabetes, and heart failure [116,117].

Vitamin D insufficiency is common in obesity and metabolic syndrome, and restoring vitamin D may ameliorate adipose dysfunction. Mechanistically, the active form (1,25-dihydroxyvitamin D_3_) acts via the vitamin D receptor (VDR) in adipocytes to reduce inflammation and modulate adipokine release [118,119]. Vitamin D suppresses pro-inflammatory cytokines (e.g., IL-6, IL-1β, IL-8, MCP-1) and even leptin in human adipocytes, while potentially boosting adiponectin, an insulin-sensitizing adipokine [118,120,121]. These anti-inflammatory and pro-metabolic effects can improve insulin sensitivity in adipose tissue. In the heart, vitamin D signaling is emerging as an important regulator of cardiac metabolism and remodeling. Cardiomyocytes and other cardiac cells express VDR, and vitamin D exerts cardioprotective effects: it dampens inflammatory cell infiltration in myocardium, reduces cardiomyocyte apoptosis, and modulates autophagy [122,123]. Vitamin D also helps preserve mitochondrial function in the heart; it promotes mitochondrial homeostasis and limits oxidative damage to proteins and lipids [124]. Consistently, vitamin D deficiency has been linked to myocardial dysfunction, whereas sufficient vitamin D may support normal contractility and prevent pathological hypertrophy [125].

Magnesium is a cofactor in hundreds of enzymes crucial for energy metabolism, including those governing glucose uptake and ATP synthesis [126]. In adipose tissue, magnesium deficiency triggers a pro-inflammatory, insulin-resistant state. Hypomagnesemia in obesity is associated with excessive reactive oxygen species (ROS) generation, mitochondrial dysfunction, and reduced ATP production in adipocytes [127]. Clinically, low magnesium correlates with greater insulin resistance, whereas higher magnesium intake is strongly associated with improved insulin sensitivity, especially in overweight individuals [128]. Magnesium repletion can enhance adipocyte glucose uptake and adipokine profiles, thereby improving metabolic homeostasis. In the cardiovascular system, magnesium plays a similarly vital role. Magnesium deficiency is known to suppress cardiac mitochondrial function and increase oxidative stress, which can impair myocardial energetics [129]. Indeed, in heart failure models, magnesium restoration improved mitochondrial ATP production and reduced oxidative damage, translating into better diastolic function [130]. Adequate magnesium thus helps maintain normal cardiac bioenergetics and electrical stability, whereas deficiency has been linked to arrhythmias, hypertrophy, and worsened outcomes in heart failure [129].

Zinc is an essential trace element that modulates antioxidant defenses and metabolic signaling. In adipose tissue, low zinc status exacerbates metabolic inflammation. Obese individuals often show suboptimal zinc levels, which are associated with aggravated insulin resistance, chronic inflammation, and heightened oxidative stress in adipose depots [131,132]. Mechanistically, zinc is required to activate antioxidant enzymes (such as Cu/Zn-superoxide dismutase) that scavenge ROS and to regulate cytokine expression [133]. Zinc sufficiency restrains adipose tissue inflammation and helps maintain healthy adipokine secretion; for example, zinc deficiency can directly contribute to an inflammatory adipose phenotype and alter leptin production in obesity [134,135]. Conversely, zinc supplementation in at-risk populations has been shown to reduce systemic inflammatory markers and improve metabolic control (e.g., glucose and lipid levels) [136,137]. In the heart, zinc is crucial for redox homeostasis and cellular survival. Zinc deficiency in cardiac tissue leads to oxidative stress, inflammation, and cell death, factors that promote cardiomyopathy and heart failure [138]. A lack of zinc can impair cardiomyocyte function by disrupting calcium handling and increasing apoptosis, whereas optimal zinc levels protect the myocardium [139]. Indeed, zinc supplementation has demonstrated protective effects against myocardial ischemia–reperfusion injury and oxidative damage [140]. Thus, maintaining zinc homeostasis is important for preventing cardiac oxidative injury and unfavorable remodeling.

Iron has a dual and context-dependent role in cardiometabolic health, as both iron overload and iron deficiency can be detrimental. In adipose tissue, excess iron accumulation is pathogenic [141]. Obesity is often characterized by iron sequestration in adipose, particularly within macrophages, which creates a pro-inflammatory environment [142]. Iron overload in adipocytes and adipose macrophages promotes local oxidative stress and triggers the release of inflammatory cytokines, contributing to insulin resistance. High iron levels in fat are also linked to adipokine dysregulation—notably, adiponectin expression is suppressed when adipose iron content is elevated [143]. This combination of elevated inflammation and lower insulin-sensitizing adipokines can exacerbate metabolic syndrome. On the other hand, in the heart, iron deficiency is a prevalent problem that impairs cardiac energy metabolism [144]. Iron is required for oxygen transport (hemoglobin/myoglobin) and for mitochondrial enzymes of oxidative phosphorylation. When iron is lacking, cardiomyocytes exhibit reduced mitochondrial respiration and ATP production, leading to diminished contractile performance [145]. Approximately 50% of patients with chronic heart failure have iron deficiency, which is associated with worse exercise capacity and outcomes [146]. Iron deficiency in the failing heart correlates with mitochondrial dysfunction, greater oxidative stress, and adverse remodeling [147]. Encouragingly, treating iron deficiency (e.g., with intravenous iron) in heart failure improves symptoms and functional status, underlining the importance of iron for cardiac metabolism [148]. Yet iron excess is equally harmful in the heart: in iron-overload conditions (such as hemochromatosis), surplus iron deposits in cardiac myocytes and mitochondria, catalyzing ROS generation and lipid peroxidation that lead to fibrosis and cardiomyopathy [149]. Importantly, micronutrient deficiencies frequently coexist in patients with metabolic syndrome and obesity, creating a state of “high-calorie malnutrition” in which energy excess paradoxically accompanies inadequate vitamin and mineral status [150,151]. Deficiencies in vitamin D, magnesium, zinc, and B-vitamins cluster together and may exert synergistic deleterious effects: concurrent vitamin D insufficiency and magnesium depletion amplify NF-κB-driven adipose inflammation, while combined zinc and selenium deficits compromise antioxidant defenses and impair mitochondrial function in both adipocytes and cardiomyocytes [152,153]. This clustering suggests that single-nutrient interventions may yield limited benefit, whereas comprehensive micronutrient repletion strategies targeting multiple deficiencies simultaneously could more effectively restore adipose-cardiac axis homeostasis and interrupt the inflammatory-metabolic feed-forward loops characteristic of cardiometabolic disease [154]. A detailed summary of the key micronutrients and their roles in regulating adipose inflammation, adipokine secretion, insulin sensitivity, and cardiac mitochondrial function is presented in Table 1.

In summary, micronutrients act as essential cofactors and modulators of adipose-cardiac communication. Vitamin D and magnesium support mitochondrial function and insulin sensitivity, while zinc maintains redox balance and adipokine homeostasis. Iron requires careful balance, as both deficiency and excess impair cardiac energetics. Addressing micronutrient status represents an accessible and often overlooked strategy for optimizing cardiometabolic health.

## 5. Macronutrients Effects on Adipose-Cardiac Metabolic Flexibility

Metabolic flexibility is the capacity to rapidly switch between carbohydrate and fat oxidation as feeding or fasting states change [155]. A healthy adipose-cardiac axis buffers nutrient flux, storing fat after meals and releasing fatty acids during fasting, while the heart adjusts its fuel use accordingly. In obesity and diabetes, this coordination breaks down: insulin-resistant adipose tissue releases fatty acids even when fed, and the heart becomes fat-reliant, impairing metabolic flexibility [156,157]. Eventually, adipose storage capacity is overwhelmed, and surplus lipids accumulate in non-adipose sites like myocardium, promoting lipotoxicity and cardiac dysfunction [158,159].

Beyond macronutrient quantity, food source quality critically influences the adipose-cardiac axis through ultra-processed foods (UPFs). Even balanced macronutrient profiles from UPF impair metabolic flexibility, as emulsifiers and additives disrupt gut microbiota composition and function. These perturbations elevate microbial production of trimethylamine *N*-oxide (TMAO), a pro-atherogenic metabolite linked to endothelial dysfunction, adipose inflammation, and cardiac lipotoxicity independent of caloric intake. Prioritizing whole-food sources thus preserves microbiota integrity and optimal nutrient signaling to adipose and cardiac tissues, enhancing overall metabolic resilience [160,161].

Carbohydrate intake acutely increases insulin, which suppresses adipose lipolysis and promotes cardiac glucose uptake, a hallmark of metabolic flexibility. In healthy individuals, a high-carbohydrate meal shifts the heart toward glucose utilization as intended [162]. However, chronic excess of refined sugars can drive insulin resistance and ectopic fat deposition that impair this flexibility. In a 24-week rat study, a fructose-enriched diet (60% of calories) induced prediabetes with early diastolic dysfunction and significant molecular remodeling of the heart [163]. Despite only mild hyperglycemia, fructose-fed rats showed mitochondrial lipid perturbations, including a wholesale rearrangement of the cardiac lipidome, defective cardiolipin remodeling, and changes in 75 cardiac proteins (many related to mitochondrial function, oxidative stress, and apoptosis) [163]. Notably, these profound changes occurred before overt oxidative damage, indicating that high sugar intake can remodel cardiac metabolism and impair function at early stages of metabolic disease [163,164]. Chronic high-carbohydrate diets, especially those rich in fructose, can overload adipose stores and spill excess fuel to the heart, impairing mitochondrial function and substrate flexibility. In contrast, reducing carbohydrate intake lowers insulin, enhances adipose lipolysis, and promotes fat oxidation. Clinical trials show that replacing carbs with protein improves insulin sensitivity and glycemic control in obese, insulin-resistant individuals [165]. Together, these findings highlight that quality and quantity of carbohydrates modulate the adipose-cardiac axis: chronic sugar excess inflicts inflexibility via adipose overload and mitochondrial tweaks in the heart, whereas controlled or lower-glycemic carb intake helps maintain insulin-sensitive fuel switching [166,167].

The heart relies heavily on fatty acids for ATP, but it remains metabolically flexible—increasing glucose oxidation when glucose is abundant. High-fat diets can undermine this flexibility by chronically elevating fatty acid supply. In insulin-sensitive individuals, a short-term isocaloric high-fat feeding can paradoxically improve insulin responsiveness (likely by lowering postprandial glycemia/insulinemia) [168]. Over the long term, however, excess dietary fat often leads to adipose expansion and systemic insulin resistance, tilting the heart into a fat-dependent, glucose-underutilizing state [157]. In diabetes, elevated circulating free fatty acids drive myocardial uptake beyond energetic needs, so the heart oxidizes more fat and less glucose, with excess fuel shunted into storage as triglycerides and harmful intermediates (e.g., diacylglycerols and ceramides) [169]. Lipid accumulation drives oxidative stress, apoptosis, and contractile dysfunction, hallmarks of lipotoxic cardiomyopathy in metabolic disease. Encouragingly, enhancing adipose fat handling can protect the heart; in an HFpEF mouse model, adipose-specific MitoNEET overexpression reduced cardiac damage despite high-fat feeding [170]. These mice, which gained weight without insulin resistance, had less cardiac fibrosis and higher myocardial SIRT3 and mitochondrial dynamism proteins compared to equally obese insulin-resistant mice [170]. Efficient adipose lipid storage prevents ectopic overflow and supports adaptive cardiac mitochondrial remodeling. Early in high-fat feeding, the heart may compensate with increased glucose oxidation, but prolonged excess leads to inflexibility and energy deficits [171]. Targeting adipose-cardiac lipid handling shows promise; in diabetic mice, the adiponectin agonist AdipoRon reduced myocardial lipid accumulation and improved insulin sensitivity and cardiac function [14]. In diabetic mice, the adiponectin receptor agonist AdipoRon reduced myocardial fatty acid, triglyceride, and ceramide accumulation while improving insulin sensitivity, inflammation, and cardiac function [169]. Mechanistically, AdipoRon stimulated AMP-activated protein kinase (AMPK) and PPARα/PGC-1α pathways in the heart, enhancing mitochondrial fatty acid oxidation and clearance of toxic lipids [169,172].

Dietary protein can indirectly modulate the adipose-heart metabolic axis through its effects on body composition, satiety, and insulin dynamics [173]. Higher-protein diets often facilitate fat loss and lean mass retention, which improves whole-body insulin sensitivity and eases the burden on both adipose tissue and the heart [174]. In clinical studies, isocaloric high-protein diets have outperformed higher-carbohydrate diets in reducing insulin resistance and glycemic variability in at-risk populations [174]. By alleviating hyperinsulinemia and aiding weight reduction, increased protein intake allows adipose tissue to shrink and become more insulin-responsive, thus lowering circulating fatty acids and toxic lipid byproducts that would otherwise strain the myocardium [175]. However, not all aspects of protein are beneficial: certain amino acids, notably branched-chain amino acids (BCAAs), are elevated in obesity and have been linked to insulin resistance when chronically high. In insulin-resistant states, BCAA breakdown in adipose tissue and liver is blunted, potentially shifting metabolic stress to the heart and muscles [176]. Overall, dietary protein supports metabolic flexibility, especially when replacing refined carbs or excess fat [177]. Protein-induced weight loss lowers leptin and often raises adiponectin, a favorable shift that enhances cardiac fuel flexibility via AMPK activation, boosting glucose use and fat oxidation while reducing apoptosis [169]. Chronic protein deficiency or muscle loss can impair metabolic health, even with controlled fat intake, highlighting the need for balanced macronutrients. Protein primarily supports the adipose-cardiac axis by enhancing insulin sensitivity and adipokine signalling, promoting efficient cardiac fuel-switching [172]. Synthesizing these macronutrient-specific effects reveals a unifying principle: the adipose-cardiac axis functions optimally when nutrient supply matches metabolic demand, and dysfunction arises when this balance is chronically disrupted. Carbohydrate excess promotes hyperinsulinemia and adipose lipogenesis; fat overconsumption saturates adipose buffering, leading to ectopic lipid deposition; and protein, while generally supportive, can contribute to dysfunction when BCAAs accumulate in insulin-resistant states. Clinically, these insights support individualized dietary approaches based on metabolic phenotype rather than rigid macronutrient prescriptions. The convergence of all three macronutrients on shared endpoints, including AMPK activity, mitochondrial function, and adipokine balance, highlights the potential for synergistic dietary interventions [155,169]. An overview of how carbohydrates, fats, and proteins differentially modulate adipose tissue function, cardiac fuel selection, and overall metabolic flexibility within the adipose–cardiac axis is provided in Table 2.

In summary, all three macronutrients influence the adipose-cardiac axis through effects on insulin signaling, lipid handling, and mitochondrial function. Carbohydrate and fat excess overwhelm adipose storage capacity, promoting ectopic lipid deposition and cardiac lipotoxicity, whereas adequate protein intake supports metabolic flexibility. Optimal macronutrient balance should be tailored to individual metabolic status to preserve adipose-cardiac coordination.

## 6. Inter-Organ Communication Beyond Adipokines: Exosomes, miRNAs, and Gut Microbiota

Inter-organ signalling from adipose tissue to the heart extends well beyond classical adipokines. Small extracellular vesicles (sEVs, often referred to as exosomes) and their non-coding RNA cargo, together with gut-microbiota-derived metabolites, form a highly dynamic communication network that transduces nutrient and inflammatory states in adipose depots into cardiac metabolic and structural responses [47,178]. Exosomes are 40–150 nm vesicles formed within multivesicular bodies and secreted by most cell types, including mature adipocytes, stromal vascular cells, and adipose-derived stem cells (ASCs). Adipose-derived sEVs carry lipids, proteins, metabolites, and a characteristic set of non-coding RNAs (miRNAs, circRNAs), whose composition changes with depot (visceral vs. subcutaneous), nutrient state (fed vs. fasted), and adipose inflammation.

Several recent studies show that visceral adipose tissue releases sEVs enriched in miRNAs that impair insulin signalling in peripheral organs and modulate inflammation; conversely, sEVs from healthy or “browned” adipose are enriched in miRNAs and proteins that promote mitochondrial biogenesis and anti-inflammatory phenotypes [47]. Adipose sEVs are taken up by cardiomyocytes, cardiac fibroblasts, and endothelial cells via endocytosis or receptor-mediated uptake; once internalized, vesicular miRNAs regulate gene networks controlling fatty acid oxidation, mitochondrial quality control, autophagy, and extracellular matrix remodelling [178].

Preclinical models implicate specific miRNAs, for example, adipose-derived miR-132/212 and miR-223, in promoting endothelial dysfunction, cardiomyocyte apoptosis, or macrophage polarization within the heart, thereby linking adipose inflammation to atherosclerosis progression and myocardial remodelling. Conversely, ASC-derived exosomes carrying cardioprotective miRNAs (e.g., miR-93, miR-22 in some models) reduce apoptosis and stimulate angiogenesis after ischemic injury. These data position sEVs as both mediators and potential therapeutic vectors [179,180].

Nutritional states reshape exosomal output: high-fat diets and obesity upregulate pro-inflammatory miRNA signatures in adipose sEVs (promoters of NF-κB and TLR4 pathways), while caloric restriction, exercise, or Mediterranean-type diets shift cargo toward mitochondrial-supportive and anti-fibrotic miRNAs [180]. Importantly, visceral adipose sEVs are often more pathogenic than subcutaneous depot sEVs in animal studies, aligning with clinical observations that visceral adiposity more strongly predicts cardiometabolic risk [181]. The gut microbiome modulates cardiac physiology indirectly through multiple metabolites, short-chain fatty acids (SCFAs), secondary bile acids, and trimethylamine-N-oxide (TMAO) being chief examples, and through effects on adipose inflammation and exosome secretion [182]. SCFAs produced by fiber fermentation improve adipose insulin sensitivity and can promote thermogenesis, whereas microbiota that favor TMAO production (from dietary choline/carnitine) correlate with adverse cardiac outcomes. There is growing evidence of crosstalk: microbiota-derived metabolites alter adipose sEV cargo and secretion rate, and adipose inflammation alters gut barrier integrity and microbial composition, creating feed-forward loops that modulate cardiac risk [183].

Mechanistically, gut microbiota modulate host exosome production through several interconnected pathways. Microbial metabolites, particularly SCFAs such as butyrate and propionate, act on adipocytes via G-protein-coupled receptors (GPR41/GPR43) and histone deacetylase inhibition, altering intracellular signaling cascades that regulate exosome biogenesis and cargo loading [184,185]. Lipopolysaccharide (LPS) from dysbiotic microbiota activates TLR4-NF-κB signaling in adipocytes, promoting release of pro-inflammatory sEVs enriched in miRNAs such as miR-34a and miR-155 that target anti-inflammatory pathways [186,187]. Dietary inputs thus exert simultaneous effects across this network: high-fiber intake promotes SCFA-producing bacteria (Bifidobacterium, Faecalibacterium), which enhance adiponectin secretion, reduce inflammatory adipokine output, and shift adipose sEV miRNA profiles toward anti-inflammatory signatures (increased miR-22, miR-146a; decreased miR-34a) [188]. Conversely, Western diets rich in saturated fat and L-carnitine favor TMAO-producing taxa and LPS translocation, suppressing adiponectin while elevating resistin, IL-6, and pro-fibrotic exosomal miRNAs [183]. These integrated signals converge on cardiomyocytes: SCFA-conditioned adipose sEVs deliver miRNAs that support mitochondrial biogenesis (via AMPK/PGC-1α) and reduce oxidative stress, whereas dysbiosis-associated sEVs carrying miR-34a inhibit SIRT1 and promote cardiomyocyte apoptosis and fibroblast activation [186,189]. TMAO itself synergizes with pro-inflammatory exosomal cargo to activate TGF-β/Smad pathways in cardiac fibroblasts, accelerating fibrosis and diastolic dysfunction [190]. This tripartite gut–adipose–heart communication network demonstrates how nutrient inputs shape microbial ecology, adipokine profiles, and sEV-mediated miRNA transfer in an integrated manner that ultimately determines cardiac metabolic flexibility and resilience.

**Figure 1:**
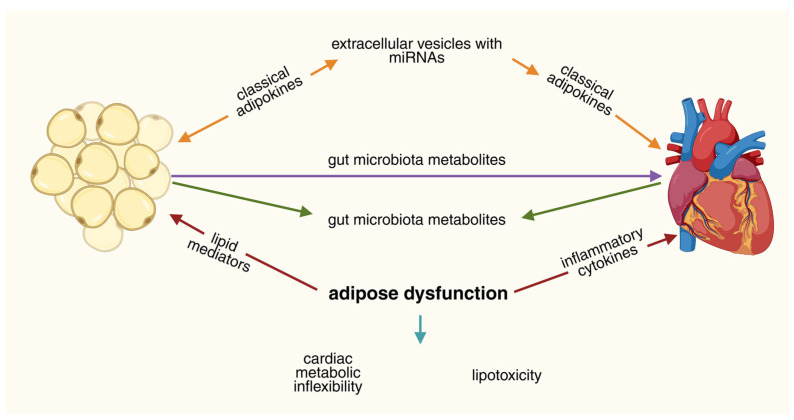
Multi-Level Adipose–Cardiac Axis Communication: Integrating Classical and Emerging Signaling Pathways. The figure illustrates the multilayered communication network between adipose tissue (left) and the heart (right). Adipose-to-heart signaling (efferent arm): Adipose tissue releases classical adipokines (adiponectin, leptin, resistin) that modulate myocardial metabolism, insulin sensitivity, and inflammatory tone. Extracellular vesicles (sEVs) carrying miRNAs (e.g., miR-34a, miR-122, miR-146a) transfer post-transcriptional regulatory signals that influence cardiomyocyte gene expression, mitochondrial function, and fibrotic pathways. Lipid mediators, including free fatty acids and ceramides, serve as metabolic substrates but in excess contribute to cardiac lipotoxicity and metabolic inflexibility. Heart-to-adipose signaling (afferent arm): The failing or stressed heart releases inflammatory cytokines (TNF-α, IL-6, IL-1β) and natriuretic peptides that act on adipose tissue to alter lipolysis, adipokine secretion, and thermogenic capacity. These cardiac-derived signals contribute to systemic inflammation and can exacerbate adipose dysfunction, creating pathological feed-forward loops. Gut microbiota as a modulatory axis: Microbiota-derived metabolites, including short-chain fatty acids (SCFAs), trimethylamine *N*-oxide (TMAO), and secondary bile acids, independently influence both adipose tissue function and cardiac responses, representing an environmental layer that intersects with host-derived signaling. Central convergence on adipose dysfunction: The intersection of these signaling layers at adipose tissue dysfunction (center) illustrates how metabolic, inflammatory, and microbiota-derived inputs converge to drive cardiac metabolic inflexibility and lipotoxicity. Overall, the figure depicts an integrated system in which classical hormones, vesicle-bound non-coding RNAs, lipid substrates, inflammatory mediators, and microbiota-derived metabolites form distinct but interconnected communication layers modulating the adipose–cardiac axis.

Adipose sEVs and their miRNA cargo are attractive biomarkers (circulating vesicles can reflect depot-specific pathology) and promising therapeutic modalities [191]. Two translational strategies are emerging: inhibit pathogenic sEV production or uptake (targeting the ESCRT machinery, neutral sphingomyelinase, or blocking key surface ligands); or engineer sEVs or synthetic nanoparticles to deliver cardioprotective miRNAs or metabolic regulators to the heart. Parallel microbiome interventions (dietary fiber, targeted pre-/probiotics, and inhibitors of microbial TMA production) aim to shift metabolite profiles and, indirectly, reprogram adipose signalling. Early preclinical successes have been reported, but human proof-of-concept trials remain limited [192]. This multi-layered adipose–cardiac communication network, integrating classical adipokines, sEV-derived miRNAs, and gut microbiota metabolites, is summarized in Figure 1.

In summary, inter-organ communication extends beyond classical adipokines to include adipose-derived extracellular vesicles carrying miRNAs and gut microbiota-derived metabolites such as SCFAs and TMAO. These emerging mediators translate nutritional and inflammatory states in adipose tissue into cardiac metabolic and structural responses, offering novel biomarker and therapeutic opportunities for cardiometabolic disease.

## 7. Biomarkers and Translational Therapeutic Targets in the Adipose-Cardiac Axis

Accurate biomarkers that capture adipose health, inter-organ signalling, and cardiac metabolic flexibility are central for translating mechanistic insights into patient care. Biomarkers relevant to the adipose-cardiac axis can be categorized according to their clinical utility: diagnostic biomarkers identify the presence or severity of adipose dysfunction and its cardiac consequences; prognostic biomarkers predict disease trajectory, adverse events, or mortality risk; and therapeutic-response biomarkers track the efficacy of nutritional, pharmacological, or lifestyle interventions. Several molecules serve dual or triple roles depending on clinical context, underscoring the integrated nature of adipose-cardiac communication. Complementing biomarkers, several translational targets have matured to the point of clinical testing or near-term clinical translation [193].

Diagnostic biomarkers enable identification and phenotyping of adipose-cardiac dysfunction. Circulating adiponectin (particularly the high-molecular-weight fraction) remains a robust inverse marker of adipose dysfunction and a predictor of improved cardiac metabolic profile; low adiponectin associates with impaired myocardial FAO and greater lipotoxicity [194]. Ceramides (distinct species, e.g., C16:0/C18:0) have emerged as reproducible lipid biomarkers of lipotoxic stress and cardiovascular risk that integrate adipose lipolytic activity and hepatic lipid handling; ceramide panels have predictive value beyond traditional lipids in multiple cohorts. Advanced imaging (cardiac MR spectroscopy for myocardial steatosis, FDG-PET or dedicated tracers for brown adipose quantification, CT/MRI-derived visceral adipose radiodensity) adds tissue-level phenotyping to circulating biomarkers and enhances the prediction of who will benefit from targeted metabolic interventions [195,196].

Prognostic biomarkers predict disease trajectory and cardiovascular outcomes. Acylcarnitine profiles, ketone body levels, and circulating mtDNA/mitochondria-derived peptides provide readouts of mitochondrial substrate flux and damage and are promising markers of cardiac metabolic flexibility [197,198]. Circulating adipose-derived sEVs carry depot-specific miRNA signatures that may serve as early indicators of pathogenic adipose signalling before overt clinical disease. Several groups have described EV-miRNA panels (including miR-34a, miR-122, and miR-192) associated with fibrosis, insulin resistance, and atherosclerotic progression. EVs have practical advantages for stability and tissue specificity, but methodological standardization and large prospective validation are outstanding needs [191]. Elevated TMAO levels independently predict major adverse cardiovascular events and heart failure progression, linking gut microbiota composition to long-term cardiac risk. Echocardiography and circulating NT-proBNP/hs-troponin remain essential for clinical endpoints [47,199].

Therapeutic-response biomarkers track the efficacy of interventions targeting the adipose-cardiac axis. Changes in adiponectin levels, HOMA-IR, and ceramide profiles following dietary interventions (Mediterranean diet, caloric restriction) or pharmacotherapy (GLP-1 agonists, SGLT2 inhibitors) provide mechanistic confirmation of treatment effects. Shifts in circulating sEV-miRNA signatures and reductions in TMAO with dietary fiber or microbiome-targeted therapies offer additional metrics for monitoring metabolic reprogramming. These biomarkers complement traditional endpoints when assessing dietary or metabolic therapies [47,199]. Several mechanistic targets have direct translational lines. Adiponectin pathway—adiponectin receptor agonists and agents increasing adiponectin expression are under preclinical/early clinical evaluation to restore cardiac mitochondrial function and reduce fibrosis [194]. Lipotoxicity axis, inhibitors of ceramide synthesis and modulators of fatty-acid oxidation (CPT1 modulators, ATGL regulators) aim to rebalance myocardial substrate handling; ceramide-lowering shows promising associations with improved outcomes in early human studies [198]. SIRT1/AMPK activation—NAD+ precursors, SIRT1 activators (resveratrol analogues), and AMPK activators remain attractive for enhancing mitochondrial biogenesis and metabolic flexibility; formulation and target specificity are active development challenges [200,201]. EV therapeutics, ASC-derived exosome products designed to deliver cardioprotective miRNAs or proteins, are in translational pipelines; scalability and safety (immunogenicity, off-target effects) remain to be resolved [202]. Microbiome-targeted approaches—dietary fiber interventions to raise SCFAs, inhibitors of microbial TMA formation, and targeted probiotic consortia are being evaluated for their capacity to lower systemic inflammation, reprogram adipose function, and reduce cardiac risk [192].

GLP-1 receptor agonists and SGLT2 inhibitors represent emerging therapeutic modalities that impact the adipose-cardiac axis beyond glycemic control. GLP-1 agonists modulate appetite and energy intake and activate key metabolic pathways, such as AMPK and mTOR, that overlap with mechanisms of calorie restriction and healthy diets. Clinically, they reduce visceral and epicardial adipose tissue volumes, thereby improving cardiac metabolism and reducing inflammation. SGLT2 inhibitors also support cardiac remodeling by inducing mild ketosis, enhancing myocardial ketone utilization, and improving metabolic flexibility, similar to ketogenic diets. These agents complement biomarker-driven strategies by targeting pathological adipose signals and promoting mitochondria [203,204,205].

A translational pathway requires combining accessible biomarkers (serum ceramides, adiponectin, EV-miRNA panels) with imaging phenotyping and randomized intervention trials that use biomarker changes as mechanistic endpoints. Critical gaps include the standardization of EV assays, prospective validation of ceramide panels across diverse populations, and well-powered human trials to determine whether biomarker-guided interventions (e.g., ceramide lowering, adiponectin agonism, or microbiome modulation) can achieve durable reductions in primary cardiovascular outcomes [198]. In summary, biomarkers reflecting adipose dysfunction (adiponectin, ceramides, sEV-miRNAs) and imaging modalities (cardiac MR spectroscopy, FDG-PET) can stratify patients for targeted interventions. Emerging therapies, including adiponectin receptor agonists, AMPK/SIRT1 activators, GLP-1 receptor agonists, SGLT2 inhibitors, and microbiome-targeted approaches, aim to restore adipose-cardiac coordination and improve cardiometabolic outcomes. Key biomarkers and emerging therapeutic targets that reflect adipose dysfunction, inter-organ crosstalk, and cardiac metabolic flexibility across obesity, diabetes, and heart failure are summarised in Table 3.

## 8. Conclusions and Future Directions

Metabolic and signaling crosstalk between adipose tissue and the heart is profoundly influenced by nutrient status. This review illustrates that macronutrients and key micronutrients can remodel the adipose-cardiac axis, promoting metabolic flexibility or driving maladaptation in obesity, diabetes, and heart failure. However, despite growing insights into how carbohydrates, fats, proteins, and vitamins/minerals (e.g., vitamin D, magnesium, zinc, selenium, and iron) affect adipokine signaling and cardiac fuel use, significant knowledge gaps remain. The interplay among multiple nutrients and the long-term consequences of dietary patterns on the adipose-heart axis remain poorly elucidated, underscoring the need for more comprehensive mechanistic studies and longitudinal clinical research [116,126,138,145,147].

A primary future direction is to decipher the complex, context-dependent effects of nutrients on inter-organ communication. For instance, how combined macronutrient excess (or restriction) and micronutrient imbalances jointly shape adipose inflammation, adipokine profiles, and cardiac substrate preference remains unclear. Advanced in vivo models and multi-omics approaches could help unravel nutrient–gene interactions and identify new mediators of adipose-cardiac signaling [117,155,156]. Emerging evidence indicates that adipose-derived exosomal microRNAs and gut microbiota metabolites convey nutritional signals to the heart, warranting further investigation [158,162,163]. Standardization of assays (e.g., for extracellular vesicle biomarkers) and large-scale validation studies are critical next steps. Several specific unmet clinical needs warrant prioritization: standardized EV-miRNA panels require consensus protocols for vesicle isolation, miRNA quantification, and normalization to enable cross-study comparisons and clinical translation [206]; longitudinal dietary intervention trials simultaneously tracking nutritional biomarkers (adiponectin, ceramides, TMAO, sEV-miRNAs) and cardiac remodeling parameters (echocardiographic indices, MR-derived steatosis and fibrosis markers) are needed to clarify whether biomarker changes precede or follow structural cardiac adaptations [207]; and head-to-head comparisons of dietary patterns using hard cardiometabolic endpoints would strengthen evidence-based prescription for specific patient phenotypes. Likewise, well-powered human trials are needed to test whether modulating these pathways—through diets or pharmaceuticals—can meaningfully improve cardiac outcomes [148,149,159,166].

From a clinical standpoint, these findings carry promising therapeutic implications. Correcting common nutrient deficiencies (such as vitamin D or iron) is an attractive strategy, given their links to myocardial dysfunction and heart failure outcomes [159,174,178,180]. Novel interventions targeting adipose-derived signals are also on the horizon; for example, adiponectin receptor agonists aim to restore cardioprotective adipokine activity, and GLP-1 receptor agonists and SGLT2 inhibitors modulate adipose tissue inflammation and enhance cardiac metabolic flexibility by reducing visceral fat and lipotoxicity while improving energy substrate utilization [169,194]. Microbiome-targeted diets or probiotics seek to reduce pro-inflammatory signaling [47,172]. Such approaches could enhance cardiac metabolic flexibility and reduce lipotoxic damage. Importantly, translating these advances into practice will require rigorous clinical trials and biomarker-guided strategies to confirm improved patient outcomes [169,193]. Bridging these gaps through future research will pave the way for nutrient-based interventions that bolster the adipose-cardiac axis and ultimately combat cardiometabolic disease.

## Figures and Tables

**Table 1:** Overview of Key Micronutrients (Vitamin D, Magnesium, Zinc, and Iron) and Their Roles in Regulating Adipose Inflammation, Adipokine Secretion, Insulin Sensitivity, and Cardiac Mitochondrial Function.

Micronutrient	Key Effects on Adipose Tissue	Key Effects on the Heart	Refs
Vitamin D	Acts via adipocyte VDR to reduce inflammation and modulate adipokine release. Suppresses IL-6, IL-1β, IL-8, and MCP-1; may increase adiponectin. Improves adipose insulin sensitivity.	VDR is expressed in cardiomyocytes and regulates remodeling and metabolism. Reduces apoptosis, inflammation, and regulates autophagy. Deficiency linked to myocardial dysfunction and pathological hypertrophy.	[118,125]
Magnesium	Deficiency induces a pro-inflammatory, insulin-resistant adipocyte state. Hypomagnesemia increases ROS and mitochondrial dysfunction and reduces ATP production. Higher Mg intake correlates with better insulin sensitivity.	Essential for ATP synthesis and mitochondrial function. Magnesium deficiency increases cardiac oxidative stress and impairs energetics. Repletion improves mitochondrial ATP production and diastolic function; deficiency is linked to arrhythmias and hypertrophy.	[126,127,128,129,130]
Zinc	Low zinc levels worsen adipose inflammation and oxidative stress. Deficiency alters leptin production and impairs healthy adipokine secretion. Supplementation reduces inflammatory markers and improves metabolic control.	Required for antioxidant defenses and cardiomyocyte survival. Zinc deficiency → oxidative stress, inflammation, impaired calcium handling, apoptosis. Supplementation protects against ischemia–reperfusion injury.	[131,132,133,134,135,136,137,138,139,140]
Iron	Excess iron accumulation in adipocytes and macrophages drives oxidative stress, inflammation, and insulin resistance. High adipose iron suppresses adiponectin, promoting metabolic dysfunction.	Iron deficiency impairs mitochondrial respiration and ATP production. Common in heart failure; predicts worse exercise capacity and outcomes. Treatment with IV iron improves symptoms; iron overload causes ROS-driven fibrosis and cardiomyopathy.	[141,142,143,144,145,146,147,148,149]

**Table 2:** Summary of the Distinct Metabolic and Functional Effects of Carbohydrates, Fats, and Proteins on Adipose Tissue Function, Cardiac Fuel Selection, and Overall Adipose–Cardiac Metabolic Flexibility.

Macronutrient	Key Effects on Adipose Tissue	Key Effects on the Heart	Refs
Carbohydrates	Acute insulin rise suppresses lipolysis and increases adipose glucose uptake. Chronic excess (refined sugars, fructose) induces insulin resistance, adipose overload, and increased adipose-to-heart fuel spillover.	High-carb meals shift myocardium toward glucose utilization (metabolic flexibility). Chronic fructose or sucrose excess leads to mitochondrial dysfunction, altered cardiac lipidome, early diastolic dysfunction.	[162,163,164,166,167]
Fats	High-fat feeding promotes adipose expansion and, over time, insulin resistance. Dysfunctional adipose fails to buffer fatty acids → overflow to the heart.	Excess fatty acid supply increases myocardial FA uptake and storage → lipotoxicity, oxidative stress, apoptosis. Protective effects when adipose fat storage is efficient (e.g., MitoNEET mice).	[157,168,169,170,171]
Proteins	High-protein diets enhance satiety, reduce fat mass, and preserve lean mass → improve insulin sensitivity. Reduced hyperinsulinemia and improved adipose responsiveness lower circulating FFA spillover.	Improved systemic metabolic control reduces myocardial lipid load and promotes healthier fuel switching. Some amino acids (e.g., BCAAs) may be detrimental when chronically elevated.	[165,169,172,173,174,175,176,177]

**Table 3:** Principal Biomarkers and Emerging Therapeutic Targets Reflecting Adipose Dysfunction, Inter-Organ Crosstalk, and Cardiac Metabolic Flexibility in Obesity, Diabetes, and Heart Failure.

Category	Biomarker/Target	Role in Adipose–Cardiac Axis	Refs
Adipokines	Adiponectin	Marker of adipose health and metabolic flexibility. Low levels are associated with impaired myocardial fatty acid oxidation, lipotoxicity, and fibrosis. Therapeutic target: adiponectin receptor agonists improve cardiac mitochondrial function and reduce inflammation.	[169,177,194]
Lipotoxicity Markers	Ceramides (C16:0, C18:0)	Reflect systemic and cardiac lipotoxic stress. Elevated in obesity/diabetes; predict cardiovascular risk beyond traditional lipids. Therapeutic target: ceramide-lowering strategies and FAO modulators.	[197,198]
Mitochondrial Stress Markers	Acylcarnitine profile, mtDNA, mitochondrial peptides	Indicate disrupted substrate oxidation and mitochondrial injury. Useful for assessing cardiac metabolic flexibility and response to interventions.	[197,198]
Extracellular Vesicles/miRNAs	Adipose-derived sEV-miRNAs	Carry depot-specific signatures reflecting adipose inflammation and metabolic dysfunction. Affect cardiac FA oxidation, autophagy, fibroblast activation, and remodeling. Therapeutic target: engineered sEVs delivering cardioprotective miRNAs.	[47,178,179,180,191]
Imaging Biomarkers	Cardiac MR spectroscopy (myocardial steatosis)	Quantifies cardiac lipid content—predicts lipotoxicity and metabolic rigidity.	[195]
	FDG-PET/metabolic imaging	Tracks cardiac glucose utilization; detects altered substrate preference in metabolic disease.	[196]
Gut–Heart Mediators	Microbiota metabolites (SCFAs, TMAO)	SCFAs improve adipose insulin sensitivity; TMAO is linked to cardiovascular risk and cardiac remodeling. Target: TMA inhibition, pre/probiotic modulation.	[182,183]
Therapeutic Pathways	Adiponectin receptor agonists	Restore mitochondrial function, reduce lipid accumulation, and enhance FA oxidation.	[169,194]
	AMPK/SIRT1 activators	Enhance mitochondrial biogenesis, improve metabolic flexibility.	[200,201]
	Exosome-based therapies	Deliver cardioprotective miRNAs to reduce apoptosis and improve remodeling.	[179,180,181,182,183,191,192]
	Microbiome-targeted therapy	Fiber, SCFA enhancers, and TMA inhibitors reduce systemic inflammation and improve adipose signaling.	[183]

## Data Availability

Not applicable.
